# Genetic polymorphisms associated with anti-malarial antibody levels in a low and unstable malaria transmission area in southern Sri Lanka

**DOI:** 10.1186/1475-2875-11-281

**Published:** 2012-08-20

**Authors:** Rajika L Dewasurendra, Prapat Suriyaphol, Sumadhya D Fernando, Richard Carter, Kirk Rockett, Patrick Corran, Dominic Kwiatkowski, Nadira D Karunaweera

**Affiliations:** 1 Department of Parasitology, Faculty of Medicine, University of Colombo, Colombo, Sri Lanka; 2 Bioinformatics and Data Management for Research, Office for Research and Development, Faculty of Medicine, Siriraj Hospital, University of Mahidol, Bangkok, Thailand; 3 University of Edinburgh, Edinburgh, UK; 4 Wellcome Trust Centre for Human Genetics, University of Oxford, Oxford, UK; 5 London School of Hygiene and Tropical Medicine, University of London, London, UK; 6 Wellcome Trust Sanger Institute,, Wellcome Trust Genome Campus,, Hinxton, Cambridge,, UK; 7 Malaria Genomic Epidemiological Network, University of Oxford, Oxford, UK

**Keywords:** Anti-malarial antibodies, Host genetic mutations, Low malaria transmission

## Abstract

**Background:**

The incidence of malaria in Sri Lanka has significantly declined in recent years. Similar trends were seen in Kataragama, a known malaria endemic location within the southern province of the country, over the past five years. This is a descriptive study of anti-malarial antibody levels and selected host genetic mutations in residents of Kataragama, under low malaria transmission conditions.

**Methods:**

Sera were collected from 1,011 individuals residing in Kataragama and anti-malarial antibodies and total IgE levels were measured by a standardized ELISA technique. Host DNA was extracted and used for genotyping of selected SNPs in known genes associated with malaria. The antibody levels were analysed in relation to the past history of malaria (during past 10 years), age, sex, the location of residence within Kataragama and selected host genetic markers.

**Results:**

A significant increase in antibodies against *Plasmodium falciparum* antigens AMA1, MSP2, NANP and *Plasmodium vivax* antigen MSP1 in individuals with past history of malaria were observed when compared to those who did not. A marked increase of anti-MSP1(*Pf*) and anti-AMA1(*Pv*) was also evident in individuals between 45–59 years (when compared to other age groups). Allele frequencies for two SNPs in genes that code for IL-13 and TRIM-5 were found to be significantly different between those who have experienced one or more malaria attacks within past 10 years and those who did not. When antibody levels were classified into a low-high binary trait, significant associations were found with four SNPs for anti-AMA1(*Pf*); two SNPs for anti-MSP1(*Pf*); eight SNPs for anti-NANP(*Pf*); three SNPs for anti-AMA1(*Pv*); seven SNPs for anti-MSP1(*Pv*); and nine SNPs for total IgE. Eleven of these SNPs with significant associations with anti-malarial antibody levels were found to be non–synonymous.

**Conclusions:**

Evidence is suggestive of an age–acquired immunity in this study population in spite of low malaria transmission levels. Several SNPs were in linkage disequilibrium and had a significant association with elevated antibody levels, suggesting that these host genetic mutations might have an individual or collective effect on inducing or/and maintaining high anti–malarial antibody levels.

## Background

Malaria used to be a major public health problem in Sri Lanka with almost 0.3 million positive cases reported 10 years ago [1]. However, a significant reduction of malaria incidence was observed after the year 2002 due to many reasons, including the modifications introduced to the National Malaria Control Programme (in 1993) to be in line with the New Global Malaria Control Strategy recommended by the World Health Organization. In this context, Sri Lanka has moved to a phase of “elimination” of malaria [2]. The number of cases reported from the Moneragala District, Sri Lanka, which was considered an endemic area for both vivax and falciparum malaria, has also dramatically reduced since 2001 (Table 1). A previous study carried out in this locality to quantify genetic and non-genetic contributions to malaria infections (by pedigree analysis of longitudinal data) has shown that host genetic factors do contribute to the variation in the frequency and intensity of clinical episodes of malaria in this population [3]. This current study was planned as a follow-up in order to investigate the profile of 6 selected anti-malarial antibodies of these residents and study its association with host genetic mutations in selected genes. 

**Table 1:** Malaria incidence reported from Moneragala District and from the study areas to Malaria Research Station (MRS) – Kataragama (1998 – 2008)

**Year**	**Malaria incidence reported from Moneragala District**			**Malaria incidence reported from the study area to MRS – Kataragama**		
	***P. v.*(+)**	***P. f.*(+)**	**Total**	***P. v.*(+)**	***P. f.*(+)**	**Total**
1998	20924	3124	24048	753	677	1430
1999	35471	7202	42673	1847	1228	3075
2000	30752	10133	40885	2802	1072	3874
2001	3246	459	3705	420	114	534
2002	624	181	805	253	24	277
2003	350	42	392	97	3	100
2004	136	15	151	0	0	0
2005	16	1	17	0	0	0
2006	1	0	1	0	0	0
2007	6	0	6	0	0	0
2008	3	1	4	0	0	0

Source of data: Anti-malaria campaign, Ministry of Healthcare and Nutrition, Sri Lanka and database maintained at MRS.

The immune status of the residents living in areas with low malaria transmission and its relationship with other demographic characteristics has been reported from elsewhere previously [4,5]. Many studies have also been conducted to evaluate the role of host genetic factors associated with serological responses. Luoni *et al.* reported the association between the T allele of the IL4-524 polymorphism and elevated antibody levels against malaria antigens in West Africa [6]. Similar results were obtained in further studies in Burkina Faso and Ghana showing association of IL4*-*589 C/T allele with increased levels of anti-*Plasmodium falciparum* IgG antibodies and total IgE levels [7,8].

This study looks at the immune status and its relationship with demographic changes and selected host genetic markers of residents in eight villages in the Moneragala district, Sri Lanka where the malaria incidence has declined steadily over the past decade.

## Methods

### Ethical clearance

Ethical clearance for this study was granted by the Ethics Review Committee, Faculty of Medicine, University of Colombo. One thousand and eleven individuals over 14 years of age and who gave written consent to participate in the study were recruited to the study. Proxy consent was obtained for the young participants (aged 14–18 years) from their parents or the guardian/s.

### Study area

This study was conducted in eight adjacent villages in Kataragama Medical Officer of Health (MOH) division in the district of Moneragala [1]. Kataragama is an area in the dry lowland coastal plains of south-east Sri Lanka, where the malaria situation is considered “unstable” and low within the last decade.

### Recruitment of individuals for the study

The number of houses in each village and number of individuals in each house were listed, based on previous census records maintained by the field research facility at the Malaria Research Station, Kataragama [1]. Each house and each individual living in that house was given a unique number for identification. The study subjects were visited during four consecutive visits to the area between December 2006 and May 2007 in order to collect the relevant data and blood samples for DNA extraction, sera and thin/thick blood smears.

### Sample and data collection

Five mL of blood was collected from all study subjects to normal tubes for serum samples and for EDTA–coated tubes for DNA extraction. Thin and thick blood smears were prepared for examination for the presence of parasites in the blood. Each tube and the corresponding slides were labeled according to the serial number given for each individual. Data on age, sex, history of previous clinical malaria during the past 10 years and information on the use of bed nets were recorded.

### Serum separation and ELISA

Serum was separated from the clotted blood samples by centrifugation (12,000 rpm for eight minutes) and analysed for six anti-malarial antibodies (ie, anti-AMA1, anti-MSP1, anti-MSP2, anti-NANP, for *P. falciparum*, anti-AMA1 and anti-MSP1 for *Plasmodium vivax*) and total IgE with the levels determined using standard ELISA technique. Fifty μL of each antigen at a dilution of 0.5 μg/mL (for AMA1 (3D7), MSP2 (3D7) and IgE) or 1 μg/mL (for MSP1 (Wellcome genotype) and NANP [(NANP)4] were coated on ELISA plates (Immulon4 ELISA plates/Dynatech) and were incubated at 4°C overnight. The plates were washed with a solution of PBS and 0.05% Tween20 (PBS/T) and 200 μL of blocking solution (2% skimmed milk in PBS/T) were added before incubating the plates for three hours in ambient temperature. Serum samples were added in duplicates after washing the incubated plates with PBS/T. These were incubated overnight at 4°C. Plates were washed in PBS/T and 50 μL of horseradish peroxidase – conjugated rabbit anti-human IgG (DAKO) diluted 1/5,000 in PBS/T were added and incubated for three hours at room temperature. The plates were again washed with PBS/T and OPD substrate solution (100 μL/well) was added and left at room temperature for 10–15 minutes for the assay to develop. 2 M H_2_SO_4_ (25 μL/well) was added to stop the reaction and the plates were read at 492 nm in an ELISA reader ( Additional file 1 OD values). The cut-off value of the assay was determined by calculating the arithmetic mean of the absorbance of negative control samples obtained from European individuals who had never been exposed to malaria and adding three standard deviations to that value (mean OD + 3SD). Using standard positive and negative controls, positive–negative threshold baseline was constructed using OD values obtained upon ELISA and was used for calculation of the observed antibody titres as described by Snyder *et al.* in 1983 [9].

### DNA extraction and genotyping

DNA was extracted from whole blood (2.5 mL) collected in to EDTA tubes using Nucleon BACC2 commercial DNA extraction kit [Gen-Probe Life Sciences, Tepnel Research Products & Services, Manchester, UK]. Five ng of gDNA was whole-genome amplified by primer-extension pre-amplification (PEP) using N15 primers (Sigma, UK) and Biotaq (Bioline, UK) polymerase as previously described by Zhang *et al.*[10]. Single nucleotide polymorphisms (SNPs) were assayed on the Sequenom® iPLEX platform according to manufacturer’s instruction using diluted PEP DNA (1:10). Genotype calls were made using the Sequenom® Typer v4.03 software [11].

### Screening for malaria infection

Blood samples were screened for malaria infection by microscopic examination of stained thin and thick blood smears by trained microscopists.

### Data analysis

Antibody levels were analysed in relation to age, gender and the history of malaria. For the genetic analysis a total of 170 SNPs in 62 genes were genotyped ( Additional File 2 SNPs). This included 65 SNPs with known associations with malaria infection/disease severity. SNPs were filtered according to the following criteria: SNPs with less than 95% genotype call, not in Hardy-Weinberg Equilibrium (p < 0.05), or monomorphic were excluded from further analysis. Individuals who had less than 95% of the SNPs genotyped were also excluded from further analysis. After quality control of SNPs and samples, 118 SNPs in 1,008 individuals were selected for further analysis.

Data were analysed using SPSS V 13.0, R software package for statistical analysis (2.6.1) and MINITAB 14.0. Kruskall–Wallis test and Mann–Whitney U tests were used to compare and study associations and Fisher’s Exact and Chi-squared tests were also used for genotype analysis. Significance of SNPs was confirmed by generation of q values using the software QVALUE (V1.0) [12]. Linkage disequilibrium (LD) plots for the 19 genes where the 23 significant SNPs were located were generated using Haploview (V4.2).

## Results

### Characteristics of the population

Ages ranged from 14 to 89 years with approximately equal numbers of males and females (514:497). Over 99% of the population belonged to one ethnic group (Sinhala) with only seven belonging to Tamil ethnicity. The majority of individuals (>95%) used bed nets. Only 18.4% of individuals (186/1,011) have had clinical malaria within the past 10 years and none within the past five years (Table 2). At the time of sample collection, blood smear examinations were negative for malaria parasites in all subjects.

**Table 2:** Demographic characteristics, history of disease and seropositivity of the study population

**Total (1,011**)	**Number (%)**
Males	514 (50.8)
Females	497 (49.2)
**Age distribution**	
14 – 29	380 (37.6)
30 – 44	307 (30.4)
45 – 59	234 (23.1)
60 – 74	71 (7.0)
>75	19 (1.9)
**Ethnicity**	
Sinhala	1,004 (99.3)
Tamil	7 (0.7)
Moor	0 (0.0)
**History of malaria within past 10 years**	
Yes	186 (18.4)
No	532 (52.6)
Cannot remember	293 (29.0)
**History of malaria within past five years**	
Yes	0 (0.0)
No	1,011 (100.0)
Cannot remember	0 (0.0)
**Bed net use in 1992/94**	
No	111 (10.9)
Yes	896 (88.6)
Cannot remember	4 (0.3)
**Bed net use last night**	
No	40 (4.0)
Yes	968 (95.7)
No response	3 (0.3)
**Seropositivity (sero-prevalence)**	
Pf_AMA1	866 (85.66)
Pf_MSP1	845 (83.58)
Pf_MSP2	856 (84.67)
Pf_NANP	919 (90.90)
Total IgE	848 (83.88)
Pv_AMA1	1,007 (99.60)
Pv_MSP1	981 (97.03)

### Results of serological investigations

Greater than 80% and >97% of participants were seropositive for *P. falciparum* and *P. vivax* respectively (Table 2). The study population was divided into three groups based on the participants’ past history of clinical malaria within the past 10 years (Table 2). Anti-AMA1 (*Pf*) (p = 0.004), anti-MSP2 (*Pf*) (p = 0.027), anti-NANP (*Pf*) (p = 0.002) and anti-MSP1 (*Pv*) (p = 0.003) levels were significantly higher in people who have had one or more clinical malaria episodes within the past 10 years, when compared to those who gave no evidence of clinical malaria during past 10 years (Table 3).

**Table 3:** Comparison of median antibody levels of people with and without recent history of malaria and between the sexes (Group A- People with no history of disease within past 10 years, Group B-People with history of malaria disease within past 10 years) 293 individuals who stated ‘did not remember’ having a malaria attack were excluded in this specific analysis

	**Median anti-malarial antibody levels and total IgE levels (Q1-Q3) (International Units per microlitre except IgE levels measured in ng/mL)**						
	**Anti-AMA1_Pf**	**Anti-MSP1_Pf**	**Anti-MSP2_Pf**	**Anti-NANP_Pf**	**Total IgE**	**Anti- AMA1_Pv**	**Anti- MSP1_Pv**
A (n = 532)	531.05	1450.15	1126.80	3390.35	4390.40	3356.15	2190.60
	(211.96-1587.54)	(589.35-3092.42)	(516.52-2893.34)	(1878.68-6233.46)	(1850.20-8572.42)	(1407.27-9834.10)	(1113.22-5902.23)
B (n = 186)	885.10	1619.40	1492.45	4460.45	4163.30	3662.70	3559.70
	(255.78-1972.92)	(603.70-3420.35)	(637.78-4450.73)	(2344.25-8624.20)	(1467.97-8258.32)	(1559.33-9297.81)	(1332.49-8794.58)
p value	0.004^*^	0.239	0.027^*^	0.002^*^	0.405	0.405	0.003^*^
Male (n = 353) (range)	592.43	1597.69	1340.89	3479.23	5719.66	3166.38	2386.78
	(242.19-1701.04)	(673.87-3395.07)	(561.36-3324.04)	(2010.22-6952.50)	(2569.56-10853.45)	(1403.19-9701-93)	(1238.26-6724.18)
Female (n = 365) (range)	624.68	1501.08	1214.67	3585.46	3298.72	3691.38	2326.02
	(200.56-1958.36)	(587.94-3349.18)	(519.17-3369.79)	(1913.57-6683.77)	(1189.41-5921.31)	(1591.25-10161.87)	(1122.44-6756.56)
p value	0.791	0.493	0.865	0.476	<0.001^*^	0.056	0.983

Q1-Q3 (1^st^ quartile-3^rd^ quartile).

Significant values [*Mann Whitney *U* test].

Total study population was categorized into five age-groups based on their ages at the time of collection of serum (Table 2). The three age groups comprised 14–29, 30–44, and 45–59 years old and were almost equally represented (37.6%; 30.4%, 23.1% respectively) while a relatively lower proportion of subjects represented the >60-year-old age group. A high level of anti-MSP1 (*Pf*) (p = 0.001) and of anti-AMA1 (*Pv*) (p < 0.001) was observed in the 45–59 year old age group (when compared to other age groups) (Table 4).

**Table 4:** Median levels of the six anti-malarial antibodies and total IgE levels in different age groups in the study population (a cross-section of the population from age >14 yrs grouped into five age groups)

	**Median anti-malarial antibody levels and total IgE levels (Q1-Q3) (International Units per microlitre except IgE levels measured in ng/mL)**						
**Age group**	**MSP1_Pf**	**MSP2_Pf**	**AMA1_Pf**	**NANP_Pf**	**IgE**	**AMA1_Pv**	**MSP1_Pv**
14-29	1319.27	1088.68	534.77	3594.79	3963.39	2766.07	2194.75
	(476.78-3107.21)	(452.85-3329.50)	(186.71-1918.41)	(1942.41-7020.00)	(1479.99-8132.72)	(1202.57-8831.96)	(939.18-7030.56)
30-44	1350.6	1250.91	593.89	3212.47	4291.16	3209.42	2264.82
	(614.17-3163.44)	(601.21-3291.47)	(203.75-1741.98)	(1890.03-6015.25)	(1880.78-8081.28)	(1598.00-8223.37)	(1245.76-5786.05)
45-59	2037.26*	1628.88	691.92	3812.24	4935.19	5174.55**	2752.59
	(948.82-4132.19)	(652.23-3847.64)	(283.74-1795.61)	(1985.26-9252.97)	(2013.94-9753.25)	(2067.67-14464.98)	(1471.60-8346.44)
60-75	1733.84	1128.77	592.54	3921.37	4469.11	3851.21	2670.22
	(889.44-3703.19)	(564.93-2823.93)	(248.00-1609.49)	(2088.93-7266.04)	(2106.46-6952.20)	(1842.01-13399.11)	(1454.50-6004.46)
>75	1729.72	1177.5	778.67	2967.53	4867.95	4677.19	1849.98
	(1287.32-3077.00)	(716.23-2667.64)	(325.45-1708.98)	(2538.34-4836.03)	(1826.49-7184.74)	(2810.53-8257.18)	(1125.00-4363.06)
p	0.001	0.372	0.406	0.477	0.33	p < 0.01	0.142

Anti-MSP1 (*Pf*)* and anti-AMA1 (*Pv*)** levels were significantly higher in the 45–59 year age group (p = 0.001, p < 0.001 respectively – p values obtained by performing Kruskal-Wallis test for association between median antibody levels of different age groups).

Q1-Q3 (1^st^ quartile-3^rd^ quartile).

The levels of all six anti-malarial antibodies were comparable between males and females (Table 3). However, the total IgE concentration between males and females were seen to be significantly different (p < 0.001) with the median concentration of total IgE in males (5,719.66 ng/mL) being significantly higher when compared to females (3,298.72 ng/mL).

Antibody levels were classified into a low-high binary trait using a cut-off of 5,000 IU/mL by ploting a histogram of antibody levels transformed in to logarithm base 10. The threshold was set at 10 for the histogram, as there was a clear separation between two peaks at this point.

**Figure 1:**
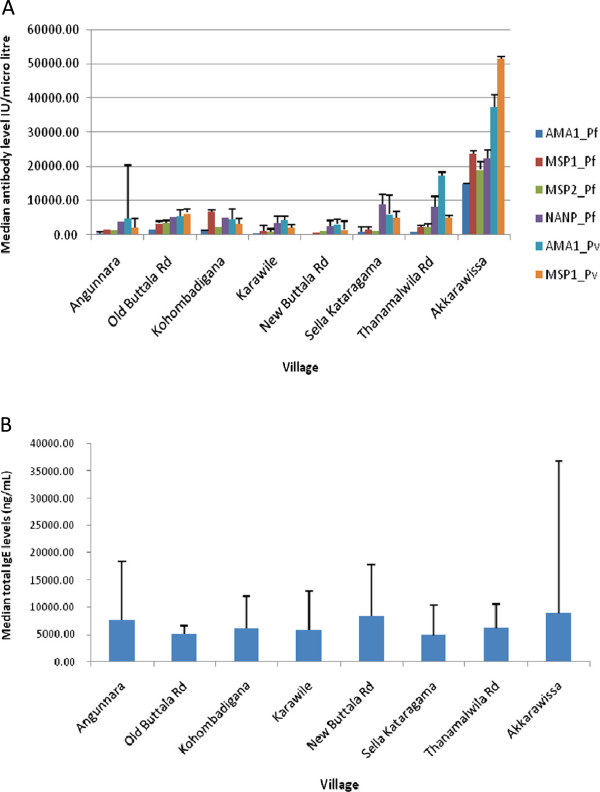
**Median of anti-malarial antibodies and total IgE levels of residents of the eight villages in Kataragama, Monereagala District included in the study: Akkarawissa, Angunnara, Karawile, Kohombadigana, New Buttala Road, Old Buttala Road, Sella Kataragama and Thanamalwila.** The residents of Akkarawissa showed higher levels of all six anti-malarial antibodies compared to the residents of other seven villages.

The residents of Akkarawissa consistently showed overall higher antibody levels compared to the residents in all other villages (Figure 1A-1B) for not only all the anti-malarial antibodies but IgE as well. These high overall levels were contributed by >60% of the village particpants who had high antibody levels (>5,000 IU/ml), in contrast to <10% of individuals from the other villages.

### Relationships of serological data and genetic data

Genetic data was analysed against the categorical classification of antibody data using a cut-off of 5,000 IU/ml to define the low and high antibody groups. Significant association was found with four SNPs and anti-AMA1(*Pf*); two SNPs and anti-MSP1(*Pf*) (none for anti-MSP2(*Pf*)); eight SNPs and anti-NANP(*Pf*); two SNPs and anti-AMA1*(Pv)*; six SNPs and anti-MSP1*(Pv)*; and seven SNPs and IgE (Table 5). Eleven SNPs out of these 28 were located in genes that code for interleukins, ie, IL4, IL10, IL13 and IL22. Most of these markers within the interleukin genes were seen to be in association with high levels of *P. falciparum* anti-malarial antibodies and five out of seven host genetic markers, which were significantly associated with high IgE levels, were located in IL4 and IL10 genes (with the other two being located in GBP7 and DERL3 genes).

**Table 5:** Function and details of identification of the significant SNPs associated with high levels of each anti-malarial antibody levels and total IgE levels

**Antibody**	**Significant SNPs**	**Chromosome (Position)**	**Gene**	**Allele associated**	**p**	**Function**
AMA1_Pf	rs25882	5 (131411460)	CSF2	T	0.039	NS
	rs1881457	5 (131992409)	IL13	T	0.047	S
	rs334	11 (5248232)	HBB	A	0.008	NS
	rs1799969	19 (10394792)	ICAM1	G	0.043	NS
MSP1_Pf	rs2706348	5 (131933709)	RAD50	G	0.039	
	rs2227478	12 (68648622)	IL22	A	0.043	S
MSP2	NONE					
NANP	rs7537937	1 (89582690)	GBP7	C	0.003	NS
	rs1800872	1 (206946407)	IL10	T	0.033	S
	rs1800871	1 (206946634)	IL10	A	0.031	S
	rs25887	5 (131416061)	CSF2	C	0.013	NS
	rs156029	5 (131532634)	P4HA2	A	0.0007	
	rs272867	5 (131681057)	SLC22A4	G	0.039	
	rs1881457	5 (131992409)	IL13	T	0.019	S
	rs334	11 (5248232)	HBB	A	0.001	NS
AMA1_Pv	rs4986790	9 (120475302)	TLR4	A	0.039	NS
	rs1801033	5 (41199959)	C6	A	0.049	NS
MSP1_Pv	rs1801274	1 (161479745)	FCGR2a	T	0.018	NS
	rs156029	5 (131532634)	P4HA2	A	0.016	
	rs848	5 (131996500)	IL13	G	0.030	S
	rs2242665	6 (31821494)	CTL4	A	0.015	NS
	rs7935564	11 (5718517)	TRIM5	A	0.025	NS
	rs10775349	16 (4079823)	ADCY9	G	0.042	S
Total IgE	rs7537937	1 (89582690)	GBP7	C	0.024	NS
	rs1518110	1 (206944861)	IL10	C	0.002	S
	rs1800872	1 (206946407)	IL10	T	0.001	S
	rs1800871	1 (206946634)	IL10	A	0.001	S
	rs2243250	5 (132009154)	IL4	C	0.023	S
	rs2243270	5 (132014109)	IL4	T	0.039	S
	rs3177244	22 (24179132)	DERL3	G	0.043	NS

NS: non-synonymous, S: synonymous.

The only interleukin-associated host genetic marker that was significantly associated with high levels of *P. vivax* antibodies was rs848 for anti-MSP1 (*Pv*), which is a coding SNP in IL13. The SNP coding for the sickle-cell variant (HbS or rs334), which is situated in HBB was significantly associated with high antibody levels against both AMA1 and NANP of *P. falciparum* antigens. It was interesting to note that the majority of SNPs were segregated on a few selected chromosomes with eight and 11 SNPs out of 28 found within genes located on chromosome 1 and chromosome 5 respectively. However, taken together, none of the SNPs had significant association with elevated levels of all tested antibodies (Chi-Squared test, Regression analysis, p < 0.05). Nine SNPs, which had significant associations with elevated antibody levels were found to be synonymous in their function (Table 4), which included all significant SNPs located in interleukin genes. However, some of the studied SNPs, e.g. those located in FCGR2a, SLC22A4, TLR4, ICAM1 and HBS genes, were non-synonymous in function (Table 5). Allele frequencies in two SNPs (rs20541 and rs7935564) in two genes, ie, IL13 and TRIM5 were found to be significantly different (p = 0.038) between those with past history of malaria (one or more clinical malaria attacks during last 10 years, i.e. malaria-susceptible individuals) and people without past history, i.e. apparently protected from malaria (Chi-Squared test p = 0.036).

Significance of allele frequencies of SNPs was further confirmed by the calculation of q values for each p value using the software QVALUE (QVALUE Version 1.0; Alan Dabney and John D Storey; pi0 =1, FDR level 0.05).

### Linkage disequilibrium

**Figure 2:**
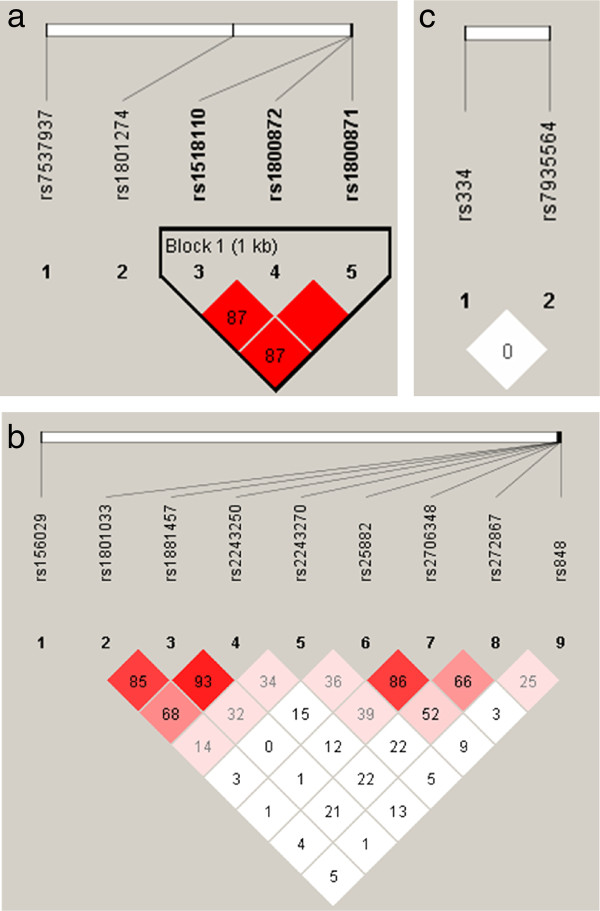
**Pair-wise linkage disequilibrium (LD) plots of the significant single nucleotide polymorphisms of Chromosome 1 (Figure** 2**a), Chromosome 5 (Figure** 2**b) and Chromosome 11 (Figure** 2**c).** The numbers in each box represents 100 x D’ value. Red squares indicate pairs of SNPs in high linkage (D’ ~ 1.00). The plots were generated using Haploview software.

Two SNPs (rs1800872 and rs1800871), which were significant for elevated levels of anti-NANP (*Pf)* and total IgE, were in linkage disequilibrium (D’ = 1.00, LOD = 334.61, r^2^ = 1.00)(Figure 2a-2c). These two SNPs were also in LD with rs1518110 (D’ = 1.00, LOD = 278.77, r^2^ = 0.87) which was significant for raised levels of both anti-NANP(*Pf*) and total IgE. In chromosome 5, high linkage disequilibrium could be seen between SNPs rs1801033 and rs1881457 (D’ = 0.858, LOD = 164.96, r^2^ = 0.646), rs18811457 and rs2243250 (D’ = 0.93, LOD = 206.85, r^2^ = 0.754), rs25882 and rs2706348 (D’ = 0.86, LOD = 146.5, r^2^ = 0.667). Similarly high LD could also be seen between rs1801033 and rs22443250 (D’ = 0.689, LOD = 107.26, r^2^ = 0.472), rs2706348 and rs272867 (D’ = 0.662, LOD = 54.79, r^2^ = 0.279).

## Discussion

The study site, Kataragama is located within the malarial-endemic zone of Sri Lanka. Although most of the participants have apparently not been exposed to malaria for over a period of 10 years due to low intensity of malaria transmission, the overall prevalence of anti-malarial antibodies were seen to be very high. This might be a result of asymptomatic infections that may prevail in the area and/or prolonged memory of immune cells/factors, which would need to be confirmed following more in-depth studies [13]. Though all study subjects were considered as un-infected, based on blood-smear examination for malaria, use of more sensitive assays, e.g. PCR, could have helped to detect the presence of a sub-microscopic parasitaemia, if present. However, all individuals showed evidence of previous exposure to malaria when screened for anti-malarial antibodies. The anti-malarial antibody levels anti-AMA1 (*Pf*), MSP2 (*Pf*), NANP (*Pf*) and MSP1 (*Pv*) were significantly higher in people who had had malaria within the last 10 years. This might be due to the fact of boosting up of the immune system of these individuals when compared to people who did not get malaria during past 10 years.

Higher levels of anti-MSP1 *(Pf*) and anti-AMA1 *(Pv)* antibodies were observed among the individuals within the age group 45–59 years compared to other age groups of the community. An increase of sero-prevalence could also be observed up to this age. Such age-related trends in anti-*P. falciparum* antibody levels have been observed by many workers in diverse population groups at high [14,15] and even at low malaria transmission levels [16]. Total IgE levels of males were significantly higher than in females in the present study. Many population studies have revealed the sexual dimorphism of total IgE levels for reasons that remain unclear [17,18]. In the present study however, the particular population is an agricultural community; the men folk spend a considerable amount of time in the fields (compared to women, who engage in fewer outdoor activities) exposing themselves to allergens and/or antigens, that induce high levels of IgE, which play an important role in allergy and are especially associated with type I hypersensitivity [19].

It was interesting to note that the residents in village Akkarawissa had notably higher antibody levels than did the residents of the other seven villages. Akkarawissa is a farming village its residents mainly focusing on “*Chena* cultivation” where they burn the patches of jungles to cultivate their crops and its people are known to spend considerable amounts of time in the cultivation areas in the middle of the jungles, compared to other villagers. It is also surrounded by foliage that gives refuge to mosquitoes and other insects. Therefore, it is likely that there is higher exposure to malaria among the residents of this particular area although there was no significant difference between the numbers of past malaria attacks as recalled by these residents when compared to others. In-depth epidemiological studies would need to be carried out to prove (or disprove) the likelihood of increase exposure to malaria by these individuals.

Twenty-three SNPs out of 169 SNPs that were tested were found to be in association with the high levels of anti–malarial antibodies (Chi squared test, p < 0.05). Polymorphism rs1801274 in FCGR2A was associated with elevated levels of anti–MSP1 (*Pv*). A study done in two ethnic groups in Mali has shown the same polymorphism that had an influence on *P. falciparum* reactive IgG levels, which was related to protection against malaria [20]. A study based in India has revealed that homozygotes for the polymorphisms rs1801274 correlated with susceptibility to severe falciparum malaria [21]. It was suggested by Parren *et al.* in 1992 that this polymorphism could affect the regulation of the IgG subclass production or turnover in humans [22]. Therefore, rs1801274 appears to be a polymorphism that is of distinct interest in malaria immunology and warrants further investigations.

A case–control study of malaria based on some ethnic groups in Sudan has revealed significant differences in genotype distribution among cases and controls of the polymorphisms rs10775349 and rs22443250 [23]. Both these SNPs were found to correlate with elevated antibodies in the study population: rs10775349 associated with anti–MSP1 for *P. vivax* and rs22443250 with total IgE levels.

Both rs1800871 and rs1800872 SNPs were significantly associated with elevated antibody levels against the NANP antigen of *P. falciparum* and were in complete linkage disequilibrium. Several investigators have reported that polymorphisms in rs1800872 IL10 promoter sequence increase the promoter activity with resultant high levels of IL10 production [24,25].

A study done in a Thai population revealed that the SNP rs1881457, which is located in the same haplotype block in 5q31-33 region with the RAD50 gene and the promoter of IL13 is significantly associated with severe falciparum malaria [26]. The present study shows that this particular SNP is associated with high levels of anti-AMA1 and anti-NANP for *P. falciparum;* these findings of the two studies regarding rs1881457 are difficult to reconcile.

## Conclusion

Age-acquired immunity to malaria (up to 59 years) seems to prevail in this low malaria transmission area of Sri Lanka; this is likely to be due to repeated exposure to infection (probably less frequent and even subclinical) over the years. Evidence point towards the presence of host-genetic variants that might have links with the generation and/or maintenance of anti-malarial antibodies that are maintained at high levels in this population inspite of the low incidence of malaria reported. No definite genetic clues could be found that favour susceptibility or protection against malaria infections. The suggested influence of the tested genetic markers on anti-malarial antibody levels could be either an individual or a combined effect since some of the markers were seen to be in linkage disequilibrium.

## Competing interests

The authors declare that they have no competing interests.

## Authors’ contributions

RLD contributed by collecting samples and clinical information, DNA extraction, data analysis and manuscript preparation. PS contributed in support for data analysis. SDF contributed in designing and supervision of the project. RC was primarily involved in designing the project at the initial stage and critical review of the manuscript. KR contributed in genotyping, sequencing and review of the manuscript. PC contributed in ELISAs. DK contributed in supervision of the project and NDK with designing, supervision of the project and support for manuscript preparation. All authors read and approved the final manuscript.

## Supplementary Material

Additional file 1 **Optical Density (OD) values for each sample tested for the six antibodies and the total IgE levels and the Corresponding antibody titres and levels OD was measured at 492 nm in an ELISA reader.** (XLS 303 kb) Click here for file

Additional file 2 **Tested Single Nucleotide Polymorphisms (SNPs) and their relevant details.** (XLS 48 kb) Click here for file
